# Pharmacokinetics and safety of liposomal bupivacaine after local infiltration in healthy Chinese adults: a phase 1 study

**DOI:** 10.1186/s12871-021-01407-5

**Published:** 2021-07-27

**Authors:** Bernard MY Cheung, Pauline Yeung Ng, Ying Liu, Manman Zhou, Vincent Yu, Julia Yang, Natalie Q. Wang

**Affiliations:** 1 Department of Medicine, University of Hong Kong, Queen Mary Hospital Pok Fu Lam, 102 Pok Fu Lam Road, Hong Kong, China; 2 Department of Adult Intensive Care, Queen Mary Hospital Pok Fu Lam, 102 Pok Fu Lam Road, Hong Kong, China; 3 Nuance Biotech Co., Ltd, Room 510, Building 2, CITIC Fortune Plaza, 9 Guangan Road, Fengtai District, Beijing, China; 4 Nuance Biotech Co., Ltd, Room 2106, Ciros Plaza, 388 Nanjing Road W, Huangpu District, Shanghai, China; 5 Pacira BioSciences, Inc., 5 Sylvan Way, Parsippany-Troy Hills, NJ USA; 6 Pacira BioSciences, Inc., Parsippany, NJ USA; 7 Present Address: Medical Global Alliance, LLC, 1330 6th Avenue, Manhattan, NY USA

**Keywords:** Tolerability, Pharmacology, Bridging, Anesthesia, Analgesia

## Abstract

**Background:**

Liposomal bupivacaine (LB) is a long-acting formulation of bupivacaine. The safety and efficacy of LB has been demonstrated across surgical procedures. However, pharmacokinetic (PK) parameters and safety of LB in the Chinese population have not been assessed.

**Methods:**

In this single-arm, single center, phase 1, open-label study, PK and safety of local infiltration with LB 266 mg were assessed in healthy Chinese adults. Eligible participants were aged 18 to 55 years with biologic parents and grandparents of Chinese ethnicity, in generally good health (i.e., no clinically significant abnormalities), and with a body mass index (BMI) 19.0 to 24.0 kg/m^2^ (inclusive) and body weight ≥ 50 kg.

**Results:**

Participants (*N* = 20) were predominantly men (80 %); mean age was 32 years; and mean BMI was 21.8 kg/m^2^. After LB administration, mean plasma levels of bupivacaine rapidly increased during the first hour and continued to increase through 24 h; plasma levels then gradually decreased through 108 h followed by a monoexponential decrease through 312 h. Geometric mean maximum plasma concentration was 170.9 ng/mL; the highest plasma bupivacaine concentration detected in any participant was 374.0 ng/mL. Twenty-two treatment-emergent adverse events were reported (mild, *n* = 21; moderate, *n* = 1).

**Conclusions:**

After single-dose administration of LB, PK measures were similar to a previously reported profile in US adults. The highest observed peak plasma concentration of bupivacaine was several-fold below the plasma concentration threshold accepted as being associated with neurotoxicity or cardiotoxicity (2000–4000 ng/mL). These data support that LB is well tolerated and safe in individuals of Chinese descent.

**Trial registration:**

NCT04158102 (ClinicalTrials.gov identifier), Date of registration: November 5, 2019.

**Supplementary Information:**

The online version contains supplementary material available at 10.1186/s12871-021-01407-5.

## Background

Pain management after a surgical procedure is important for patient recovery [1]. Poor pain control is generally associated with adverse outcomes, such as complications, delayed healing, longer lengths of hospital stays, and development of chronic postsurgical pain [2–4]. In China, a survey of 124 postsurgical patients found that 70 % of respondents reported moderate to severe postsurgical pain after urology or hepatobiliary surgical procedures; these participants were also significantly more likely to report sleep disturbances and mental distress (e.g., irritability, anxiety, depression) than those reporting mild pain [5]. Therefore, effective postsurgical pain management strategies are important for mental and physical health.

In China, patient-controlled analgesia with opioids and acute analgesics (with or without opioids) are commonly used methods for pain management [5]. Although large, systematic reviews of pain management regimens used in China are limited [5, 6], systematic analyses of particular cities or regions have been conducted [7, 8]. In Shandong Province, an analysis of 625 hospitals found that patient-controlled analgesia and continuous infusion were the most frequently used methods of postsurgical analgesia and that fentanyl was the most frequently used analgesic drug [7]. In Nanjing (a developed city in mainland China), opioid consumption steadily increased from 2011 to 2016 and was 1.5-fold higher than consumption rates reported in Hong Kong [8]. Despite this increase, evidence suggests that some patients in China may be averse to using opioids for pain management after a surgical procedure. For example, a retrospective cohort study found that one of 253 patients undergoing head and neck surgical procedures at the University of Hong Kong filled an opioid prescription 6 days after the procedure [9]. A survey of 124 Chinese postsurgical patients also found that ~ 31 % reported either reluctance or objection to using opioids for postsurgical pain management after urology or hepatobiliary surgical procedures [5]. Systemic opioid exposure is associated with opioid-related adverse events (AEs; e.g., nausea, vomiting), and postsurgical opioid use may increase risk for persistent opioid use [10, 11], which could potentially contribute to reported reluctance. Indeed, a survey at a Chinese teaching hospital found that 40 % of patients who refused pain medications were concerned about the potential for addiction and AEs associated with opioids [6].

Multimodal pain management strategies involving at least two drugs utilizing different mechanisms of action can be used to reduce postsurgical pain and minimize opioid use [2]. In China, an analysis of 625 hospitals in Shandong Province reported that 36 % of institutions had adopted multimodal analgesia approaches [7]. Local infiltration with anesthetic agents into the surgical site can be used as part of multimodal analgesia, which can be further enhanced by delivering a long-acting local anesthetic to extend the duration of its therapeutic effect [12]. Liposomal bupivacaine (LB) is a long-acting formulation of bupivacaine that consists of multivesicular liposomes that slowly release bupivacaine over time and has been used in multimodal pain management regimens [13, 14]. LB is indicated for local infiltration in patients 6 years of age and older and for interscalene brachial plexus nerve block in adults by the United States Food and Drug Administration [15]; LB is also approved for brachial plexus nerve block, femoral nerve block, and field blocks in adults by the European Medicines Agency [16]. Several studies conducted in the United States have demonstrated that LB can effectively reduce pain when it is administered in several surgical procedures, including hysterectomy [17, 18], hepatectomy/liver donation [19–21], donor nephrectomy [22], abdominal wall reconstruction [23], and colorectal surgical procedures [24]. Previous studies have demonstrated that plasma bupivacaine concentrations after administration of LB generally exhibit a characteristic biphasic pattern with a small initial peak within 2 to 4 h of administration followed by a second peak occurring 12 to 36 h later as bupivacaine is released from multivesicular liposomes [13, 25]. LB and bupivacaine dosing is not bioequivalent, and up to 3-fold higher dosing of LB (266 mg) via infiltration has been shown to exhibit comparable peak plasma bupivacaine concentrations to 100 mg bupivacaine hydrochloride but with a longer release profile [25]. Importantly, previous pharmacokinetics (PK) studies have demonstrated that plasma bupivacaine concentrations after administration of LB remain well below thresholds associated with toxicity (2000 to 4000 ng/mL) [25–30].

Ethnic differences in drug metabolism can impact PK parameters and underlying genetic variation, which may ultimately cause variability in treatment responses [31, 32]. Of note, a key enzyme in bupivacaine metabolism, human cryptochrome P450 isoform CYP3A4, exhibits interindividual variability that has been largely attributed to genetic control [33, 34]. CYP3A4 polymorphisms have been extensively evaluated in various ethnic populations, with differences in allelic frequencies observed between Chinese populations compared with other populations [35]. Because the use of LB in the Chinese population has not yet been assessed, the objectives of this phase 1, open-label study were to evaluate the PK and safety of LB in healthy Chinese participants.

## Methods

### Study design

From November 7, 2019, to December 23, 2019, a single-arm, phase 1, open-label study was conducted at the University of Hong Kong to assess PK and overall safety of LB 266 mg in healthy Chinese adults (ClinicalTrials.gov identifier: NCT04158102; registration date 11/08/2019). Prior to screening, the study site obtained institutional review board approval from the Clinical Trials Centre of University of Hong Kong (The University of Hong Kong/Hospital Authority Hong Kong West Cluster IRB) that complied with the International Council for Harmonisation of Technical Requirements for Pharmaceuticals for Human Use Good Clinical Practice and/or the Hong Kong Department of Health and respective research independent ethics committees/institutional review boards. All participants provided written informed consent before any study-specific screening procedures.

### Patient eligibility

Eligible participants were Chinese adults aged 18 to 55 years with biologic parents and grandparents of Chinese ethnicity, in generally good health (i.e., no clinically significant comorbidities), a body mass index (BMI) between 19.0 and 24.0 kg/m^2^ (inclusive), and body weight ≥ 50 kg at screening and baseline. Select exclusion criteria included if participants were pregnant or of childbearing potential and were unable to use contraception; were sexually active men who did not agree to use contraception and did not agree to withhold sperm donation until 90 days after study drug administration; had concomitant, clinically significant diseases that would interfere with the study (determined by investigator’s discretion); were a study-site employee or immediate family member of an employee involved in the study; or had hypersensitivity/iatrogenic reaction to amide-type local anesthetics, abnormal findings on physical examination, or laboratory values of clinical significance.

### Study procedures

One day prior to drug administration, participants underwent baseline assessments, including medical/surgical history, demographics, physical examination, vital signs, clinical laboratory studies, breath alcohol screening, urinalysis, and human chorionic gonadotropin testing (women of childbearing potential). Eligible patients were then admitted to the study site. One day after baseline assessments (Day 1), participants received a subcutaneous injection of LB 266 mg/20 mL (manufactured by Pacira Limited, a wholly owned subsidiary of Pacira BioSciences, Inc.; EXPAREL® lot number 19-4052) via local infiltration into the superficial subcutaneous tissue of the left or right flank. All injections of LB were performed using a moving-needle technique in a 5-⨯-5-cm area designated for infiltration, with four 5-mL aliquots of LB administered in 5-mL syringes using 25-gauge or larger bore needles in a fan-like fashion from a different corner toward the center of the square. Participants remained in the clinic until Day 6 for postdose PK and safety assessments; additional PK and safety assessments occurred on Days 8, 10, and 14.

### Endpoints and assessments

This study aimed to evaluate the PK and safety of LB in healthy Chinese adults. PK measures of LB included maximum concentration (C_max_), area under the plasma concentration-time curve (AUC) from time 0 to last collection time after study drug administration (AUC_0 − last_) and extrapolated to infinity after drug administration (AUC_0−∞_), time to reach maximum plasma concentration (T_max_), half-life (t_1/2_), and clearance rate (CL/F). To determine plasma concentrations of bupivacaine, venous blood samples were collected from either arm at 18 timepoints: 0.5, 1, 2, 4, 8, 12, 24, 36, 48, 60, 72, 84, 96, 108, 120, 168, 216, and 312 h after administration of LB. Blood samples were centrifuged at 1200* g* for 10 min within 1 h of collection and stored at a nominal temperature of − 20 °C until analysis. The analytic method was validated at ABS Laboratories (York, United Kingdom). Samples (50 µL) of human plasma (dipotassium EDTA) containing the analyte and internal standard were extracted using a liquid-liquid extraction procedure. The extracted samples were analyzed by high performance liquid chromatography interfaced with an AB/MDS Sciex 4000 mass spectrometer. Positive ions were monitored in the multiple reaction monitoring mode. Quantification was by peak area ratio. Applied Biosystems Analyst 1.6.1 was used for peak integration and for calculation of concentrations.

Safety endpoints were change from baseline to prespecified timepoints for vital sign data (blood pressure, pulse, tympanic temperature) 0.5 h prior to receiving LB and at the 18 timepoints also used for PK measures, clinical laboratory tests on Day 14, and 12-lead electrocardiography on Day 14. AEs, serious AEs, and treatment-emergent AEs (TEAEs) were also monitored through Day 21. TEAEs were defined as any AE with onset on or after Day 1 but before Day 21 or any preexisting medical condition that worsened in intensity between Days 1 and 21.

### Statistical analysis

Sample size was considered appropriate for characterizing the PK profile of bupivacaine after LB administration in healthy Chinese adults. Participants who received the study drug and provided sufficient samples for PK assessments and had no significant protocol deviations were included in the PK analysis. All participants who received the study drugs were included in the safety analysis.

PK parameters were estimated using noncompartmental analysis (Phoenix WinNonlin version 8.2 (Princeton, NJ). Summary statistics (geometric mean, coefficient of variation, arithmetic mean, standard deviation [SD], median, and range) were performed with SAS, version 9.4 (SAS Institute, Cary, NC). Number of AEs was recorded and percentage of participants with AEs was calculated. Descriptive statistics were calculated for continuous data and categorical data were tabulated.

## Results

### Patient disposition and baseline characteristics

A total of 39 individuals were screened; of these, 10 participants were excluded at screening, 9 were held in reserve, and 20 were treated. All 20 treated participants completed the study. Participants were predominantly men (80 %); mean age was 32.2 years and mean body mass index was 21.8 kg/m^2^ (Table 1).

**Table 1:** Demographics and baseline characteristics

Variable	Participants
Age, mean (SD), y	32.2 (10.1)
Male, n (%)	16 (80)
Height, mean (SD), cm	170.6 (9.7)
Weight, mean (SD), kg	63.3 (7.7)
Body mass index, mean (SD), kg/m^2^	21.8 (1.3)

*SD* standard deviation

### PK analysis

**Fig. 1:**
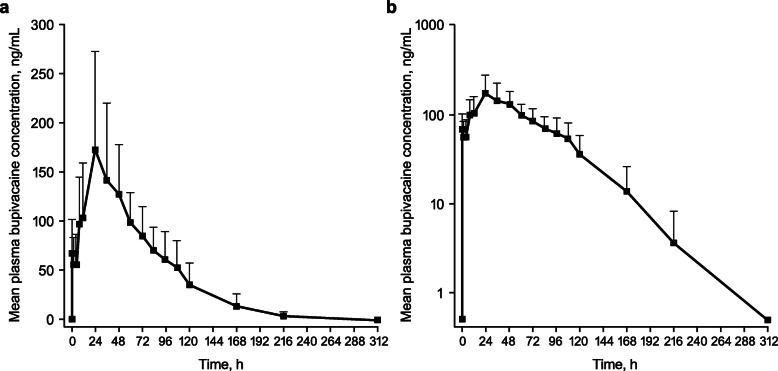
Mean plasma bupivacaine concentrations over time on (A) linear and (B) semilogarithmic scales. Error bars indicate standard deviation

After administration of LB via local infiltration, mean plasma levels of bupivacaine increased rapidly during the first hour and then continued to gradually rise until ~ 24 h. Thereafter, plasma bupivacaine levels gradually decreased until 108 h after LB administration, followed by a rapid decrease in a monoexponential manner through 312 h (Fig. 1). The geometric means of AUC_0 − last_ and AUC_0−∞_ were comparable (12,420.4 and 12,686.0 h*ng/mL, respectively; Table 2). Median T_max_ occurred at 35 h, reflecting sustained plasma bupivacaine levels after LB administration. Geometric mean C_max_ was 170.9 ng/mL, and the maximum plasma bupivacaine concentration detected in any participant was 374.0 ng/mL. Weight-normalized PK parameters can be found in Additional File 1.

**Table 2:** Pharmacokinetic Parameters of Bupivacaine

Parameter	AUC_0 − last_, h*ng/mL	AUC_0−∞_, h*ng/mL	t_1/2_, h	CL/F, mL/h	C_max_, ng/mL	T_max_, h
Mean (SD)	12,891.5 (3589.9)	13,171.3 (3675.4)	28.4 (10.4)	21,784.9 (6248.7)	189.9 (92.5)	39.9 (21.4)
Geometric mean	12,420.4	12,686.0	26.7	20,968.0	170.9	35.7
CV, %	27.9	27.9	36.7	28.7	48.7	53.7
Median (min-max)	12,260.2 (7505.3–19,332.1)	12,556.4 (7649.1–19,418.2)	26.7 (15.3–50.4)	21,189.3 (13,698.5–34,775.1)	150.0 (70.2–374.0)	35.0 (23.5-104.1)

*AUC* area under the plasma-concentration-time curve; *AUC*_*0−last*_ AUC from time 0 to last collection time after study drug administration; *AUC*_*0−∞*_ AUC from time 0 to infinity; *CL/F* clearance rate; C_*max*_ maximum concentration; *CV* coefficient of variation; *max* maximum; *min* minimum, *SD* standard deviation; *t*_*1/2*_ half-life; *T*_*max*_ time to reach maximum plasma concentration

### Safety

Fifteen participants (75 % of 20 participants included in safety analysis) experienced 22 TEAEs, and most of these events were of mild severity (*n* = 21; Table 3). One TEAE of moderate severity (gastroenteritis) was reported, which resolved by end of study. TEAEs reported by at least one participant included injection-site reaction (13 [65 %]), influenza-like illness (runny nose, cough, sore throat, fever [n = 3 {15 %}]), vessel puncture-site bruise, gastroenteritis, tonsillitis, headache, oropharyngeal pain, and rash (n = 1 [5 %] each). One TEAE of mild rash was considered possibly treatment related. No serious AEs or TEAEs leading to discontinuation or death were reported.

**Table 3:** Summary of TEAEs

Adverse event	Participant, n (%)	Event, n
Any	15 (75)	22
Any TEAE	15 (75)	22
Mild	15 (75)	21
Moderate	1 (5)	1
Any TRAE	1 (5)	1
Mild	1 (5)	1

*TEAE* treatment-emergent adverse event; *TRAE* treatment-related adverse event

## Discussion

Administration of 266 mg of LB via local infiltration resulted in sustained plasma bupivacaine levels in healthy Chinese adults. Mean bupivacaine concentrations rapidly increased in the first hour and then continued to gradually rise ~ 24 h after administration; thereafter, plasma bupivacaine levels gradually decreased for ~ 4 days after administration followed by rapid decrease in a monoexponential manner over the remaining ~ 9 days of observation. TEAEs were predominantly mild, with only one TEAE (mild rash) determined by the investigator to be possibly related to LB. Collectively, these data support that LB is safe and well tolerated in adults of Chinese descent.

The PK profile of bupivacaine in Chinese adults was comparable with that of the US population [36]. The prior study conducted in the United States predominantly included African American (70 %) and White (30 %) volunteers. In addition, mean C_max_ after a single dose of 266 mg LB in the present study (189.9 ng/mL) was comparable with the mean C_max_ (129.0 ng/mL) reported in another PK study of healthy US adults receiving a single injection of the same dose of LB [36]. However, the present analysis of Chinese adults observed peak concentrations of plasma bupivacaine ~ 11 h later than the analysis of US adults (median T_max_ of ~ 35 and ~ 24 h, respectively) [36]. Because the Americas have a much higher prevalence of overweight and obesity than the Asia Pacific region [37], it is possible that differences in BMI contributed to the differences in LB PK parameters observed here. This difference may also be partly explained by procedural differences in drug administration: LB was administered over a 25-cm^2^ area in the present study, whereas LB was administered over a 100-cm^2^ area in the study of US adults [36]. Concentrating LB over a smaller area in the present study may have led to delayed peak bupivacaine release with slightly higher concentrations.

Such procedural characteristics may be relevant when considering application of LB in a surgical setting. A review of clinical studies, which included three studies reporting administration of 266 mg LB locally via wound infiltration, found numerically higher mean C_max_ after inguinal hernia repair (365 ng/mL), total knee arthroplasty (340 ng/mL), and hemorrhoidectomy (867 ng/mL) compared with the results of the present study (190 ng/mL) [25]. Across all surgery types, a second peak in plasma bupivacaine was detected ~ 12 to 36 h after administration. These PK profile differences between healthy volunteers and surgical patients could be informative for future studies designed to evaluate efficacy and safety of LB in the surgical setting for Chinese adults.

The safety profile in the present analysis is consistent with findings in the analysis of PK of LB in US adults. Both analyses of US and Chinese adults observed injection-site reaction as a frequently reported AE and that most AEs were mild or moderate, with no reported instances of severe AEs [36]. Furthermore, plasma concentrations of bupivacaine observed during the present study (geometric mean C_max_ of 171 ng/mL; C_max_ of 374.0 ng/mL) were several-fold below the plasma concentration thresholds potentially associated with neurotoxicity and cardiotoxicity (2000 and 4000 ng/mL, respectively) [30, 36, 38]. Together, these data support a favorable safety profile of LB 266 mg administered via local infiltration in adult patients of Chinese descent.

There were several study limitations. First, patients were eligible for the study if they were in good health and had no significant comorbidities, thereby limiting generalizability of findings from this study to individuals with poor health, comorbidities, or both. For example, individuals with hepatic disease may have impaired local anesthetic metabolism and are at risk for systemic toxicity after receiving LB [15]. Second, this study was a single-dose study that lacked a comparator group (e.g., other formulations of bupivacaine, such as bupivacaine hydrochloride). Third, this study also utilized a single batch of LB; however, because of quality control measures, use of a single drug batch is unlikely to impact the generalizability of the study findings. Fourth, potential differences in body weight between regions may have hindered direct comparisons between a previous LB PK study performed in the United States [36] and the current study; body weight data were not available from the previous US study to facilitate comparison of weight-normalized PK parameters between studies. However, we have provided body weight–normalized PK parameters (Additional File 1) for easier comparison to future studies in different populations that may vary in body weight from the current study sample. Ultimately, future studies comparing LB with other anesthetics with consideration for optimal dose and patients with comorbidities will be important for establishing safety and efficacy of LB in relevant patient populations in China.

## Conclusions

The PK and safety profiles of LB in healthy Chinese adults was consistent with findings from healthy US adults. These data provide support that LB is safe and well tolerated in adults of Chinese descent.

## Supplementary information

**Additional file 1:**

## Data Availability

The datasets used and/or analyzed during the current study are available from the corresponding author on reasonable request.

## Abbreviations

AE: Adverse event
AUC: Area under the curve
BMI: Body mass index
CL/F: Clearance rate
C_max_: Maximum concentration
CV: Coefficient of variation
LB: Liposomal bupivacaine
PK: Pharmacokinetic
SD: Standard deviation
t_1/2_: Half-life
T_max_: Time to reach maximum plasma concentration
TRAE: Treatment-related adverse event
