# Supplementary material for: Pharmacokinetics and safety of liposomal bupivacaine after local infiltration in healthy Chinese adults: a phase 1 study
Source: BMC Anesthesiol. 2021 Jul 27;21:197. doi: 10.1186/s12871-021-01407-5 (PMC8314475; doi:10.1186/s12871-021-01407-5)
Supplement: Supplementary file 1 — Additional file 1: [file 12871_2021_1407_MOESM1_ESM.docx]

**Pharmacokinetics and Safety of Liposomal Bupivacaine After Local Infiltration in Healthy Chinese Adults: A Phase 1 Study**

Bernard MY Cheung, Pauline Yeung Ng, Ying Liu, Manman Zhou, Vincent Yu, Julia Yang, Natalie Q. Wang

| **Table.** Weight-Normalized Pharmacokinetic Parameters of Bupivacaine | | | | | | |  |
| --- | --- | --- | --- | --- | --- | --- | --- |
| **Parameter** | **AUC_0-last_, (h*ng/mL)/(mg/kg)** | **AUC_0-∞_, (h*ng/mL)/(mg/kg)** | **t_1/2_, h** | **CL/F,**  **mL/h/kg** | **C_max_, (ng/mL)/(mg/kg)** | **T_max_, h** |  |
| Mean (SD) | 3016.3  (745.9) | 3084.7  (785.9) | 28.4  (10.4) | 342.7  (79.3) | 43.6  (18.4) | 39.9  (21.4) |  |
| Geometric mean | 2934.8 | 2997.6 | 26.7 | 333.6 | 40.4 | 35.7 |  |
| CV, % | 24.7 | 25.5 | 36.7 | 23.1 | 42.1 | 53.7 |  |
| Median  (min-max) | 2896.3  (2092.6-4895.6) | 2943.5  (2113.0-5161.5) | 26.7  (15.3-50.4) | 339.7  (193.7-473.3) | 38.8  (20.2-83.9) | 35.0  (23.5-104.1) |  |
| AUC, area under the plasma-concentration-time curve; AUC_0-last_, AUC from time 0 to last collection time after study drug administration; AUC_0-∞_, AUC from time 0 to infinity; CL/F, clearance rate; C_max_, maximum concentration; CV, coefficient of variation; max, maximum; min, minimum, SD, standard deviation; t_1/2_, half-life; T_max_, time to reach maximum plasma concentration. | | | | | | |  |
